# Complicated Case of a Giant Bladder Stone and Forgotten Double-J (DJ) Stent in an Otherwise Healthy Elderly Patient: A Case Report

**DOI:** 10.7759/cureus.60498

**Published:** 2024-05-17

**Authors:** Abdulaziz H Khushaym, Adeel A Khan, Noora O Aljeeran, Ibrahim M AlAlhareth, Mohamed A Rafie

**Affiliations:** 1 Medical School, Arabian Gulf University, Manama, BHR; 2 Urology, King Hamad University Hospital, Manama, BHR; 3 General Surgery, King Hamad University Hospital, Manama, BHR

**Keywords:** forgotten dj stent, postoperative complications, lithotripsy, seizure, giant bladder stone

## Abstract

Giant bladder stones, defined as stones weighing over 100 g and/or measuring more than 4 cm in diameter, are relatively uncommon compared to other types of urinary tract stones. This patient, an 85-year-old male with an unknown medical history, initially presented with urinary incontinence and hematuria. Radiological findings revealed a large prostate, a forgotten left renal double-J (DJ) stent for more than 20 years with an encrusted bladder stone, and additional calculi in the lower pole of the left kidney. The patient underwent laser cystolithotripsy, but due to the complexity of the case, a second procedure was scheduled. Following the second procedure, the patient experienced a generalized tonic-clonic seizure and subsequent loss of consciousness, which was attributed to hyponatremia. The patient received appropriate management to correct hyponatremia and antiepileptic medication to control the seizure. The patient's condition eventually improved and he was discharged home with prescribed medications and follow-up appointments. This case emphasizes the potential complications of giant bladder stones and a forgotten DJ stent in an 85-year-old male patient as a rare consequence following such a rare presentation.

## Introduction

Giant bladder stones, defined as stones weighing over 100 g and/or measuring more than 4 cm in diameter, are rare occurrences in the urinary tract [1]. They can present with a wide range of symptoms, including lower abdominal pain, dysuria, gross hematuria, and urinary retention [2].

Double-J (DJ) stents, commonly used in genitourinary procedures [3], have been improved by recent advancements in stent design and materials. However, complications associated with these stents such as infection, hematuria, encrustation, and stone formation have become more common and more severe with their use [4]. The most challenging complication associated with ureteral stents is encrusted and retained ureteral stents. There have been reports of complications such as irritative voiding symptoms, urinary tract obstruction, loss of renal function, serious infection, and even death. Severe encrustation can make simple office endoscopic removal difficult, necessitating surgical removal and treatment of any accompanying encrustation stones [5].

Other general possible postoperative complications of stone management might occur, including an electrolyte imbalance. Hyponatremia is a common electrolyte abnormality characterized by low levels of sodium in the blood [6]. It can occur due to various underlying causes, including excessive fluid intake, diuretic use, renal dysfunction, hormonal imbalances, and certain medical conditions [7]. The clinical presentation of hyponatremia can range from mild symptoms, such as nausea and headache, to severe manifestations, including seizures, altered mental status, and coma [8]. Prompt recognition and appropriate management are crucial to prevent complications and optimize patient outcomes [8].

## Case presentation

**Figure 1:**
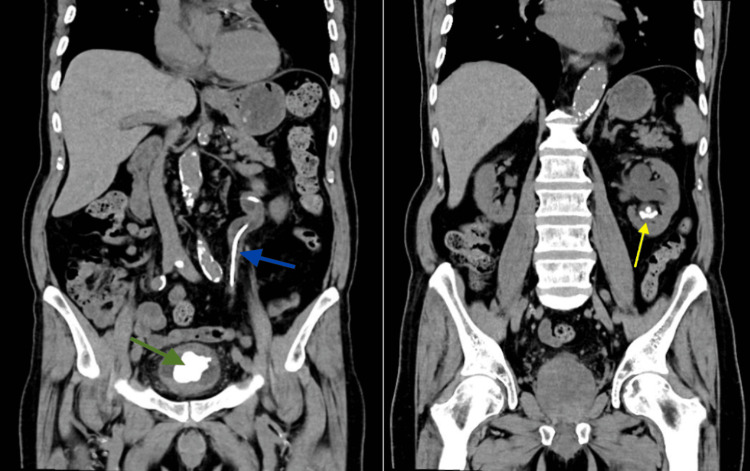
A non-contrast computed tomography for the ureter and urinary bladder (CT-KUB) Showing the bladder stone (green arrow) and the double J-stent (blue arrow) in the left image and the renal stone (yellow arrow) in the right image

**Figure 2:**
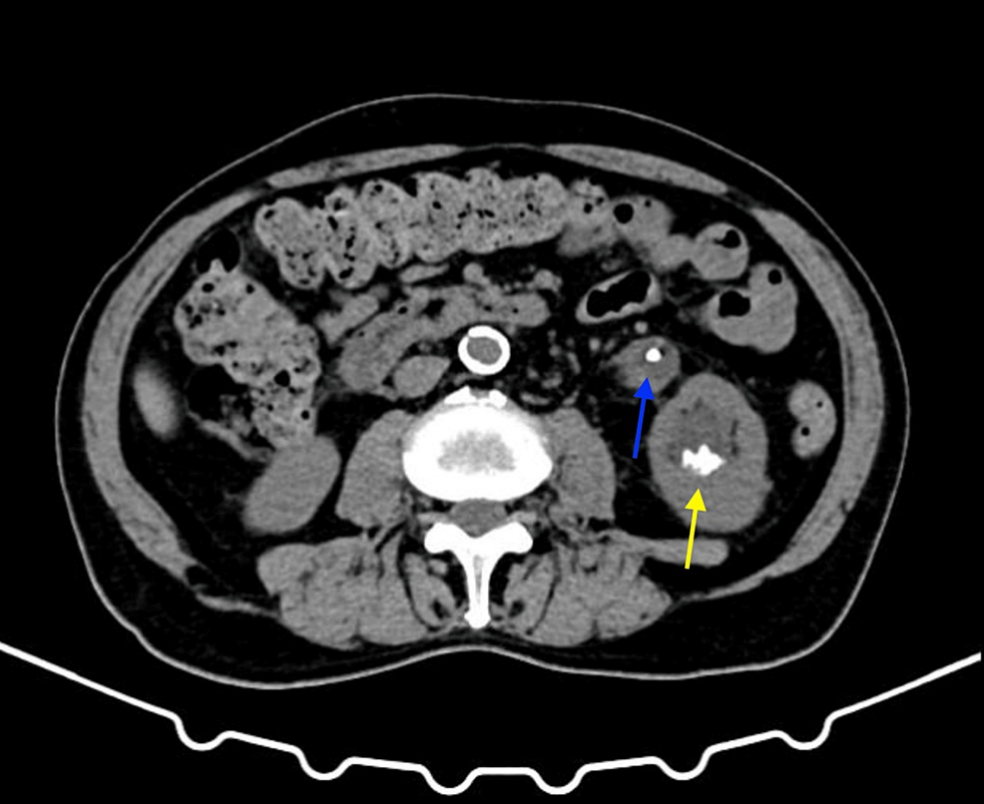
A non-contrast computed tomography for the ureter and urinary bladder (CT-KUB) Axial section showing both the left double-J stent (blue arrow) and the lower pole left renal stone (yellow arrow)

An 85-year-old male patient with an unknown medical history presented at the urology clinic with a picture of cystitis, complaining of urinary urge incontinence, hematuria, and dysuria with no other voiding or outlet obstruction symptoms. He had undergone ultrasonography of the kidney, ureter, and urinary bladder (KUB), showing a large prostate measuring 6.0 x 5.6 x 5.4 cm (volume: 90 cc). A left renal double-J (DJ) stent was noted, and although the patient couldn't recall when it was inserted, it has likely been present for more than 20 years. The stent's proximal end was located in the left proximal ureter, and the distal end was in the urinary bladder. A large bladder stone measuring up to 5.6 x 3.2 x 3.5 cm was also noted in the distal end of the encrusted left renal double-J stent in the urinary bladder. A non-contrast computed tomography for the ureter and urinary bladder (CT-KUB) was done (Figures 1, 2) and showed the bladder stone, the forgotten encrusted double-J stent, and multiple lower pole stones with an aggregate size of 22 mm.

On the day of the procedure, he received IV ceftriaxone, and a cystoscopy was performed, which revealed an enlarged, occlusive, and vascular prostate with an inflamed appearance of the bladder and a very large bladder stone on the bladder coil of the forgotten left DJ stent. A 365-micron Holimum-Yttrium-Garnet (Ho-YAG) laser fiber was used to fragment approximately two-thirds of the stone. An attempt was made to remove the DJ stent under X-ray guidance but was unsuccessful. Due to the presence of lower pole renal stones, the stuck encrusted DJ stent (Figure 2), and the length of the procedure (approximately 240 minutes), the patient was scheduled for a second elective procedure. He made an uneventful recovery and was subsequently discharged on the second postoperative day.

The patient was then scheduled for the second surgery four weeks following his first procedure. The preoperative electrolyte panel, including serum sodium, potassium, and chloride, and renal function test (urea and creatinine) were normal (Table 1). The patient underwent cystoscopy with cystolithotripsy (irrigation height was 50 cm of water), a left flexible ureteroscope, lasertripsy of lower pole renal stones, and the removal of an encrusted DJ stent. A left ureteric catheter was also inserted. Complete clearance of the bladder was achieved, along with the successful fragmentation of lower pole stones. The immediate postoperative course was uneventful with no post-obstructive diuresis. Serum electrolytes and renal function were normal on day one postoperatively (Table 1).

**Table 1:** Electrolyte panel and renal function test (pre and postoperative)

Preoperative electrolyte panel		
Parameter	Finding	Reference range
Sodium (Na)	142	(135 to145 mEq/L)
Potassium (K)	4.3	(3.5 to 5.5 mEq/L)
Chloride (Cl)	106	(96 to 106 mEq/L)
Preoperative renal function test		
Urea (blood urea nitrogen, BUN)	6.8	(2.5 to 10.7 mmol/L)
Creatinine (Cr)	86.1	(61.9 to 114.9 µmol/L)
Postoperative electrolyte panel		
Sodium (Na)	140	(135 to145 mEq/L)
Potassium (K)	4.2	(3.5 to 5.5 mEq/L)
Chloride (Cl)	105	(96 to 106 mEq/L)
Postoperative renal function test		
Urea (BUN)	8.1	(2.5 to 10.7 mmol/L)
Creatinine (Cr)	98.1	(61.9 to 114.9 µmol/L)

The patient on day two postoperatively had no complaints; his input-output charting was recorded as 800 ml and 400 ml, respectively. The catheter was then removed, and he voided three times with no retention or diuresis. The patient was assigned for discharge by the evening of the same day; however, while he was walking to the bathroom by himself and upon returning and sitting down, he suddenly developed uncontrollable jerking movements of his head and limbs with no preceding symptoms and subsequently lost consciousness for about two minutes. The patient regained consciousness spontaneously. He appeared awake but confused and complained of a headache with no difficulty of speech, muscle weakness, bowel and bladder incontinence, or any other postictal symptoms.

The initial diagnosis was made as a self-limiting episode of generalized tonic-clonic seizures. A loading dose of 2 g of IV levetiracetam was administered and monitored closely. Laboratory investigations were conducted immediately (Table 2), including serum calcium, serum magnesium, serum electrolyte panel, and renal function tests (RFTs), which showed acute hyponatremia of 126 mEq/L (normal range 135-145 mEq/L); however, the patient denied excessive fluid intake before the episode

**Table 2:** Laboratory investigations after the seizure episode

Parameter	Finding	Reference range
Sodium (Na)	126 mEq/L	(135 to145 mEq/L)
Potassium (K)	4.3 mEq/L	(3.5 to 5.5 mEq/L)
Chloride (Cl)	105 mEq/L	(96 to 106 mEq/L)
Urea (blood urea nitrogen, BUN)	8.9 mmol/L	(2.5 to 10.7 mmol/L)
Creatinine (Cr)	80.1 µmol/L	(61.9 to 114.9 µmol/L)
Calcium (Ca)	1.99 mmol/L	(2.2 to 2.7 mmol/L).
Magnesium (Mg)	0.68 mmol/L	(0.7 to 0.9 mmol/L)

A chest X-ray (CXR), electrocardiogram (ECG), and non-contrast computed tomography (CT) were performed and showed no significant abnormalities. A thorough investigation failed to detect any neurological cause of the seizure, and his electrolyte panel was monitored every six hours. Two days later, the patient was reviewed and was conscious and euvolemic. His mental status had improved, and he scored 15/15 on the Glasgow Coma Scale (GCS). He was alert and oriented to time, place, and people. There were no new neurological deficits, and no other symptoms of hyponatremia, such as vomiting, further seizures, headaches, or confusion, were noted. The patient was then subsequently discharged home. He continues to be followed up in the clinic and is recovering well.

## Discussion

The diagnosis of giant bladder stones can be challenging due to their varied presentation, which ranges from an asymptomatic patient to experiencing lower abdominal pain, dysuria, gross hematuria, and urinary retention [2]. Plain abdominal X-rays, abdominal ultrasounds, and computed tomography (CT) scans are commonly used diagnostic methods. When it comes to detecting bladder stones, ultrasound scans of the bladder have been reported to have a sensitivity ranging from 20% to 83% and a specificity ranging from 98% to 100% [1]. The insertion of ureteral stents is a significant tool in the management of numerous urologic procedures. However, ureteral stent encrustation can result in morbidity due to infection, ureteral obstruction, and stent fragmentation [9].

The formation of bladder stones generally requires the presence of predisposing factors such as bladder outlet obstruction, neurogenic bladder, chronic bacteriuria, the presence of intravesical foreign bodies, bladder diverticula, and, rarely, an upper urinary tract stone. In men, an additional common factor contributing to the formation of these stones is benign prostatic enlargement [10]. Foreign bodies, such as forgotten double-J stents or slings that inadvertently pass through the bladder, can become encrusted and act as a nidus for stone formation [11]. The presence of large bladder stones and encrustations can significantly worsen the patient's condition and lead to renal dysfunction. Although the occurrence of large bladder stones is relatively rare, the severity of complications increases with the duration of the encrustation of the stent [11].

The safe duration for keeping a stent without complications has not been fully determined, but surpassing the planned duration or neglecting its removal is deemed unsafe and potentially life-threatening [12]. The duration of the ureteral stent's presence emerged as the most significant factor in the development of encrustation, with other risk factors including metabolic or congenital disorders, stone disease, bacterial colonization, chemotherapy, pregnancy, and chronic renal failure [13].

In our situation, there were numerous risk factors for stent encrustation. The stent was neglected and forgotten about for 20 years because he was not aware that it had been inserted during the initial surgery. His limited understanding of healthcare and lack of knowledge resulted in poor patient compliance and low health literacy, which further increased the chances of stents being retained for extended periods [13].

The treatment of bladder stones generally depends on factors such as the size, location, density, and number of stones [14]. Conservative management involves adjusting the urine pH to above 6.5 for uric acid stones through urine alkalization. Extracorporeal shock wave lithotripsy (ESWT) is an alternative treatment, particularly suitable for patients with a high risk of anesthesia, those who are anxious about anesthesia or endoscopic procedures, and patients who have difficulty with positioning. However, extracorporeal shock wave lithotripsy (ESWT) is not recommended for larger stones (>2 cm), like in our case, as it has shown limited effectiveness [15].

Surgical options for removing bladder stones are either transurethral lithotripsy or open surgery, although almost all giant bladder stones reported in the literature are treated with open cystotomies [15]. By considering the patient's age, the urgency of the case, and the co-morbidity, cystoscopic lithotripsy is a preferable method in such a population compared to open vesicolithotomy. It is a minimally invasive procedure that results in shorter hospital stays and reduced overall costs [16]. The advantage of avoiding surgical wounds and scars makes endoscopic removal of bladder stones more acceptable to patients [16]. Although minor complications such as hemorrhagia and fever are more commonly reported with endoscopy, some literature considers the efficiency of transurethral lithotripsy low, as the bladder stones are large and most patients have associated severe urinary tract infections. In addition, postoperative complications and a long operation time. Open surgery for removing complete stones has shorter operation times [17].

Hyponatremia can occur due to various underlying causes, including excessive fluid intake, diuretic use, renal dysfunction, hormonal imbalances, and certain medical conditions [7]. In the absence of all other identified contributors in our case, the most likely contributor is the left flexible ureteroscope and laser lithotripsy of the lower pole renal stones. Previous research has demonstrated a strong correlation between hyponatremia during hospitalization and unfavorable clinical consequences [7].

In a recent study focusing on long-term renal prognosis in the field of urology, researchers aimed to determine the association between new-onset postoperative hyponatremia and clinical outcomes [18]. They analyzed patients who underwent bladder, prostate, ureter, and kidney surgeries and investigated the incidence of postoperative hyponatremia in each category. The study found that the occurrence of hyponatremia varied significantly across different surgery types. Kidney operations had the highest incidence of hyponatremia (27%) while bladder (12.5%), prostate (12.3%), and ureter surgeries (10.9%) showed similar incidences. Notably, transurethral resection of the prostate (TUR-P) (8.1%), cystolithotripsy (4.8%), and cystectomy (18.9%) also displayed varying rates of hyponatremia. Furthermore, this study examined perioperative characteristics and found that new-onset postoperative hyponatremia was associated with longer surgical and anesthesia times, similar to our case.

## Conclusions

This case reports the rare occurrence of giant bladder stones that developed due to a neglected DJ stent over a very long period (over 20 years) and the potential complications associated with a forgotten DJ stent and encrustation. It also highlights the challenges of dealing with postoperative complications. Moreover, it emphasizes the imperative to reduce the duration of stent placement, as forgotten stents can have severe long-term consequences. Conducting further research on biodegradable stents is essential, as they have the potential to eliminate the need for a second treatment and the risk of stent retention, particularly in resource-limited countries. This could not only lead to improved clinical outcomes but also provide financial advantages.

The establishment of a national stent registry where all stent placements are registered and reminders are issued to providers to contact patients who need stent removal has been suggested by some surgeons as a way to lower the rate of forgotten stents leading to encrustation. Close monitoring of the stent during placement, removal once it is no longer required, and regular replacement when its prolonged use is needed are crucial. To minimize the possibility of stent and post-management complications, it is also important to maintain a high fluid intake, promptly assess any clinical concerns, and actively treat infections, all of which should be used to reduce the risk of complications. Considering all of this, it is imperative to remove stents as soon as possible after they have completed their intended function to avoid complications and long-term morbidity.
